# Enhanced Photon Extraction through Optimized Waveguide Geometry for Zincblende InAsP/InP Nanowire Quantum Dots Emitting in the Telecom Range

**DOI:** 10.1021/acsanm.5c04842

**Published:** 2026-01-14

**Authors:** Giada Bucci, Tomasz Gzyl, Anna Musiał, Valentina Zannier, Fabio Beltram, Wojciech Rudno-Rudziński, Grzegorz Sęk, Lucia Sorba

**Affiliations:** † NEST, Istituto Nanoscienze-CNR and Scuola Normale Superiore, 56127 Pisa, Italy; ‡ Department of Experimental Physics, 49567 Wrocław University of Science and Technology, 50-370 Wrocław, Poland

**Keywords:** quantum dots, nanowires, zincblende crystal phase, SAE-VLS, finite-difference time-domain simulations

## Abstract

InAs_*x*_P_1–*x*_ quantum dots (QDs) embedded in InP nanowires (NWs) have recently emerged as a promising platform, offering good control over QD size, composition, and density through Au-catalyzed vapor–liquid–solid (VLS) growth. A unique advantage of this approach is the possibility of directly growing a waveguide around the QD, exploiting precise control of NW radial growth. Usually, InAs_*x*_P_1–*x*_ NW-QDs are grown along the <111> direction with a wurtzite (WZ) crystal phase, where waveguides are typically realized using selective-area epitaxy combined with VLS (SAE-VLS), requiring preparation and prepatterning of the substrates. In the case of growth along the <100> direction, the growth of defect-free zincblende InAs_*x*_P_1–*x*_ NW-QDs occurs at larger catalyst nanoparticle diameter compared to the WZ counterpart, with tunable emission over the telecom bands. Here, we show that in this system, efficient InP waveguides can be realized around the QDs without the need for SAE-VLS, solely by balancing axial and radial growth contributions during the NW growth. Employing the finite-difference time-domain simulations to optimize the NW-QD geometries allows us to experimentally investigate the interrelation between the growth parameters and the waveguide morphology. Microphotoluminescence measurements of the optimized structures confirm their improved emission properties and one order of magnitude enhanced QD emission intensity in the telecom range.

## Graphical abstract

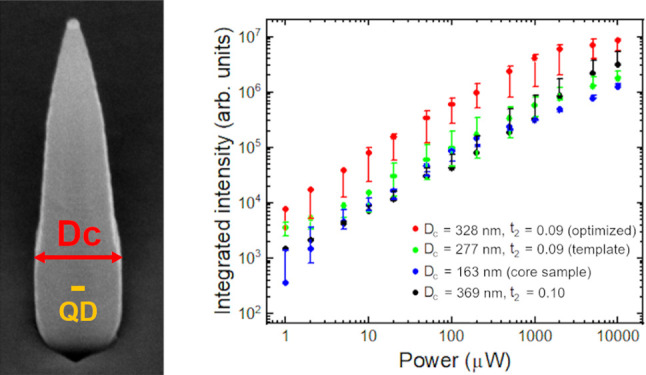

## Introduction

Semiconductor quantum dots (QDs) emitting in the telecom bands represent highly attractive light sources, as the commonly employed optical fibers exhibit minimal transmission loss and dispersion in this wavelength range.^1,2^ Moreover, single photon emission enabled by quantum confinement in QDs is fundamental for emerging quantum communication technologies and optical computing applications.^3–5^ Several material systems have been investigated to achieve telecom-band emission,^6^ among which InAs_*x*_P_1–*x*_ QDs embedded in an InP matrix stand out due to their tunable direct band gap in the telecom range without the need for strain engineering and their compatibility with mature InP-based photonic technologies.^7,8^ Different approaches have been developed for the epitaxial growth of such QDs, including strain-driven Stranski–Krastanov growth^9,10^ and droplet epitaxy.^11,12^ Recently, III–V semiconductor nanowires (NWs) have emerged as a particularly promising platform.^13–22^ Their unique geometry enables the integration of highly lattice-mismatched materials without the formation of dislocations at the interface while also offering a high degree of control over QD size, shape, and composition via growth parameter-tuning. In addition, multiple QDs can be incorporated in a single NW perfectly arranged on top of each other, with independent control of their distances along the NW axis and thickness, as well as composition, which opens a way to precisely controlled engineering of the coupling between the dots.^23^ NWs are typically obtained with vapor–liquid–solid (VLS) growth catalyzed by metal nanoparticles, most commonly gold. In the metal-assisted VLS growth, the NW diameter and density are defined by the size and density of the catalyst droplets, which also allow tuning of the QD planar dimensions and density. In addition, in situ engineering of the NW crystal phase is possible by properly choosing the substrate orientation and growth conditions, enabling further control of the QD features. Another key advantage of NW-QD systems is the possibility of direct waveguide integration. By carefully balancing axial and radial growth contributions during the NW growth, an InP shell can be epitaxially grown around the QD, realizing a waveguide with emitter perfectly positioned with respect to the propagating mode without the need of deterministic fabrication techniques based on emission imaging nor any postgrowth processing.^24^ However, realizing a waveguide employing VLS growth is nontrivial since both axial and radial growth of the NW proceed simultaneously and respond differently to the growth parameters. A common approach in the literature is to use selective-area epitaxy combined with the VLS growth (SAE-VLS).^25^ In this approach, by patterning an oxide mask with lithographically defined openings and positioning a catalyst nanoparticle at the hole centers, the NW growth is confined in the radial direction by the mask openings, enabling the formation of efficient NW antennas. First a NW core containing the QD with the diameter of the catalyst nanoparticle is grown, while in a second step, the growth conditions are tuned to realize an InP shell for the waveguiding of the QD emission. While this technique is interesting for the deterministic positioning of the QDs, it has proven effective in enhancing photon extraction and led to the achievement of many milestones;^14,18,20,21,26,27^ it however requires a pregrowth substrate fabrication and precise control of catalyst size with lithography. Another group has demonstrated the realization without prepatterning of NW antennas in InP NWs by employing a Si(111) substrate, controlling the balance between axial and radial growth in a heteroepitaxial environment.^28^ These approaches have been mainly explored in InP NWs with a wurtzite (WZ) crystal phase, grown along the <111> direction. For this growth direction and crystal phase, avoiding crystal defects demands catalyst nanoparticles of diameters below 20 nm^29^ for the VLS growth of the NW core and thus the subsequent defect-free growth of the InP shell. Such a small catalyst dimension imposes additional challenges in the fabrication process. A possible approach to avoid the presence of crystal defects for a wide range of catalyst nanoparticle diameters is to grow the NWs along the <100> direction with a zincblende (ZB) crystal phase. The growth of QDs with a ZB crystal phase is beneficial for both crystal phase purity and easiness in reaching longer emission wavelengths. In fact, different crystal symmetry results in a different band structure and, thus, in different optical features. In addition, ZB InP and InAs materials have smaller energy bandgaps than their WZ counterparts, so it is easier to reach longer emission wavelengths. ZB InAs_*x*_P_1–*x*_ QDs in InP NWs have demonstrated emission which is tunable across the telecom bands up to 1450 nm^30^ and a crystal phase which is defect-free also for catalyst nanoparticles with 30 nm diameters. However, efficient light extraction remains a key challenge for these QDs, requiring the design and realization of an integrated NW waveguide.

In this work, we address this challenge by investigating the controlled growth of InP waveguides around ZB InAs_*x*_P_1–*x*_ QDs, without the need for pregrowth fabrication steps for the SAE-VLS. Finite-difference time-domain (FDTD) simulations are employed to design the optimal InP shell morphology with constraints based on the experimentally observed NW morphology and to correlate optical performance of the structure with different waveguide parameters. By tuning the growth parameters to balance axial and radial growth, we demonstrate the realization of an optimized waveguide geometry to enhance the photon extraction efficiency (EE) of the ZB NW-QD. With microphotoluminescence (μ-PL) measurements, we investigate the dependence of the measured signal intensity on the waveguide parameters.

## Experimental Section

The NW-QD samples in this work are grown epitaxially employing Au-assisted vapor–liquid–solid growth in a Riber-21 chemical beam epitaxy (CBE) system. As gaseous metalorganic precursors, trimethylindium (TMIn), pre-cracked *tert*-butyl phosphine (TBP), and *tert*-butyl arsine (TBAs) are employed, with line pressures measured as a reference for the fluxes introduced in the chamber. The substrate temperature is measured by means of an optical pyrometer with an accuracy of ±5 °C. The substrates are made of Fe-doped InP(100) with commercial water solution gold colloids of 30 nm (BBInternational EM.GCnn) drop casted on the surface to serve as a catalyst for the VLS growth. Prior to the catalyst deposition, SiO_2_ markers are fabricated with optical lithography to facilitate the navigation on the substrate during the optical measurements and allow multiple measurements on the same NW. Prior to the growth, the substrates are outgassed at 300 °C on a heating stage to get rid of the moisture. After the growth, the samples are imaged from both the top view and 45°-tilted view employing a Zeiss Merlin scanning electron microscope operating at 5 kV to check the NW morphology.

FDTD numerical simulations are performed in commercial Ansys Lumerical software.^31^ The NW geometry optimization was performed for 1.50 μm, which is in the emission range of the currently available samples. First, in order to find the preliminary value of the NW diameter providing optimal confinement for single mode propagation along the NW, an infinitely long, untapered, square-based InP NW is modeled. QD is simulated as a point source electric dipole placed on the NW *z* axis and polarized in the *x* direction. Perfectly Matched Layer absorbing boundary conditions are set for the computational domain. Planar monitors are placed around the NW, normal to *x*, *y*, and *z* directions to determine how much of the dipole emission could be collected in these directions. Power transmitted through the monitors is then divided by the Purcell factor to obtain the spontaneous emission rates into fundamental waveguide mode Γ_11_ and leaky modes γ, which constitute the radiation losses. Coupling efficiency into a guided optical mode, known as the β factor, is calculated as β = Γ_11_/(Γ_11_ + γ). In the next step, a realistic 3D, finite-length, tapered NW with square cross-section, placed on the InP substrate is modeled. In order to reduce the simulation volume, suitable symmetric boundary conditions are set. The gold droplet on top of the NW was calculated to cause negligible changes due to its subwavelength size; thus, it is not included in further simulations. The extraction efficiency upward is determined as previously by calculating Purcell-factor normalized transmission through a planar monitor above the NW but also took into account different numerical apertures of collection optics, which are implemented by cutting out corresponding areas from the far-field projection.

To characterize the optical properties of the NW-QDs, μ-PL measurements are performed at 10 K. The samples are cooled in a closed-cycle continuous-flow liquid-helium system. A continuous wave 640 nm semiconductor laser is used for nonresonant optical excitation. Laser beam is focused onto the sample with a N.A. = 0.4 microscope objective providing diffraction limited spatial resolution of 1 μm. The detection system consists of a nitrogen cooled multichannel array InGaAs detector and a monochromator with 0.32 m focal length, equipped with a 150 grooves/mm grating blazed for 1200 nm. Overall, the spectral resolution of the experimental setup is estimated to be 0.87 meV, which is sufficient for the PL line width as observed experimentally.

## Results

### NW Growth Protocol and Morphology Evolution

The main focus of this work is the optimization of the InP shell grown around a ZB InAs_*x*_P_1–*x*_ NW-QD, with the aim of realizing an efficient waveguide. Unlike the majority of previous studies that rely on SAE-VLS,^26^ here we demonstrate that optimized InP shells can be obtained without any pregrowth substrate patterning and SAE growth, directly through the tuning of growth parameters. This approach not only simplifies the fabrication process but also allows us to systematically explore how the shell morphology depends on the balance between axial and radial growth.

The initial step of the growth protocol, common to all of the investigated samples, is the fabrication of a template NW “core”, which serves as the host for the QD and as the basis for the subsequent waveguide growth (Figure 1a). Since this core is not the focus of this work, we only briefly summarize its growth here, while details can be found in our previous work.^30^ Briefly, starting from a bare InP(100) substrate with a low density of 30 nm diameter Au colloids deposited (see Experimental Section): (i) an initial InP segment of ∼450 nm is grown at (390 ± 5) °C with TMIn and TBP line pressures of 0.3 and 0.6 Torr, respectively; (ii) a 150 s-long growth interruption is performed, while TBAs and TBP are adjusted to 0.5 and 0.4 Torr, respectively, and the temperature is linearly decreased by 20 °C; (iii) a 10 nm-height InAs_0.88_P_0.12_ QD is then grown by reintroducing the TMIn in the chamber together with TBAs; (iv) finally, an InP top segment is grown at the same temperature by interrupting TBAs supply and restoring TBP to 0.6 Torr. The resulting NW core has a defect-free ZB crystal structure and an average length of (1140 ± 20) nm and embeds an InAs_0.88_P_0.12_ QD, with an average height of (10 ± 2) nm and free of structural defects. The QD has an average planar dimension of (48 ± 4) nm measured from the TEM images, as discussed in detail in our previous work.^30^ Importantly, the choice of these QD features enables, for the first time, the realization of emission in the third telecom window from ZB InAs_*x*_P_1–*x*_ NW-QDs, as will be discussed later. The typical NW density in our samples is ∼10^6^ NWs/cm^2^ (corresponding to 10^–2^ NWs/μm^2^) and the substrate contains SiO_2_ alignment markers, which allows for investigation of single-NWs during the μ-PL measurements. The NW core acts as the starting point for our systematic study of the InP shell growth (second step in the scheme of Figure 1a), where we investigate how the shell morphology depends on the growth conditions and, in turn, how this determines the optical response of the NW-QD system. Figure 1b–e present 45°-titled scanning electron microscopy (SEM) images showing the evolution of the NW morphology with increasing NW total length *L*_tot_, averaged among the measured NWs of each sample: (b) (290 ± 60) nm, (c) (690 ± 130) nm, (d) (1140 ± 20) nm, and (e) (1450 ± 90) nm. In particular, panel (d) shows a representative NW “core”, while panel (e) displays an example of a NW-QD with an InP shell grown for 50 min under TMIn and TBP line pressures of 0.4 and 1.6 Torr.

**Figure 1:**
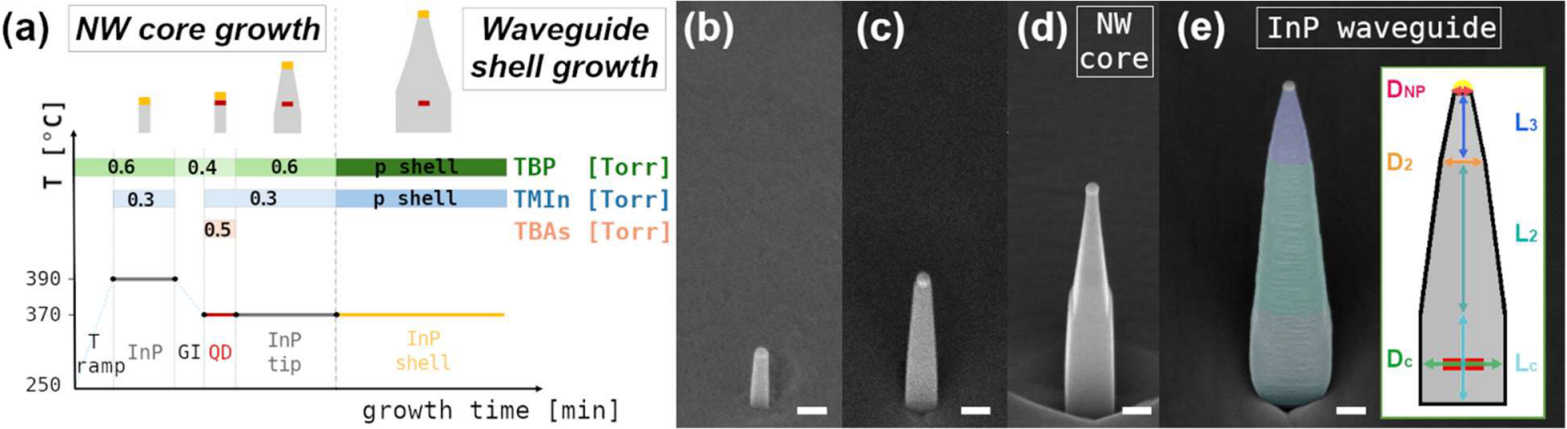
(a) Scheme of the growth protocol employed in our sample, divided into the NW core growth and the InP shell waveguide growth. For each growth step, the TBP, TMIn, and TBAs line pressures (in Torr) are reported in the green, blue, and orange boxes, respectively. A schematic of the NW growth is also reported at the graphic top; 45°-tilted side view SEM images of a representative NW with different lengths. In particular, the average total length of the ensemble is (b) (290 ± 60) nm; (c) (690 ± 130) nm; (d) (1140 ± 20) nm; and (e) (1446 ± 94) nm. Here, the three different segments of the NWs are highlighted with fake colors, and in the inset, a schematic of the NW morphology with its relevant parameters is represented. Scale bars in the SEM images correspond to 200 nm.

While we will discuss in the following the precise InP shell morphology dependence on the growth conditions, now we focus on the general shape of the InP shell. In particular, from panel (e), it can be observed that the NW shows a characteristic three-segment morphology, highlighted in false colors in the SEM image and schematically illustrated in the inset of panel (e). The first segment at the NW bottom, hereafter referred to as the “cuboid segment”, is an untapered portion of the NW with a length *L*_c_ and a constant side length *D*_c_. The second segment has a length *L*_2_, with a base side length *D*_c_ and a top side length *D*_2_. The third segment, directly below the catalyst nanoparticle, has a length *L*_3_, a base side length *D*_2_, and a top side length determined by the In–Au alloy catalyst nanoparticle, *D*_NP_ = (48 ± 4) nm. This morphology reflects the nontrivial interplay between simultaneous axial and radial growth of the {110} and {100} NW side facets. Some attempts at modeling the radial and axial growth rates to understand the different NW morphologies observed experimentally have already been done in the literature.^32–36^ Although modeling the growth dynamics of our samples is beyond the scope of this paper, we can speculate that the three-segment morphology arises from the competition between axial and radial growth at different NW heights. The untapered cuboid segment has been reported in InP NWs^28,37^ and in other materials,^36^ and, as modeled in ref 35, it has been attributed to a NW region located farther from the catalyst than the typical diffusion length of In adatoms on the sidewalls, so that the adatoms impinging on these sidewalls nucleate there, giving radial growth. The second and third tapered segments correspond to the NW regions where adatoms impinging on the sidewalls partly migrate to the catalyst (contributing to the axial growth) and partly nucleate on the sidewalls (contributing to the radial growth of the InP shell). The presence of two different taperings for the second and third segments suggests a complex growth dynamic in which the balance between the axial and radial contributions depends on the NW height. Moreover, a variation of the sidewall facets along the height of ZB InP NWs grown in the <100> direction,^38^ which also shows different partial polarities based on the tapering angle, may further influence the nucleation probability^39^ and adatom diffusion length^32^ for the two segments. By tuning the growth conditions, it should be possible to modify the relative contribution of the axial and radial growth, thereby tailoring the NW morphology. However, given the intrinsic correlation between the two growth mechanisms, the engineering of the NW morphology is nontrivial and requires a systematic study of the growth conditions on the NW geometric parameters, which is shown in the following.

### Finite-Difference Time-Domain (FDTD) Modeling

Based on the experimentally observed morphology of our NWs, we used this geometry as the input for FDTD simulations. These simulations allowed us to identify the optimal parameters—i.e., *L*_c_, *L*_2_, *L*_3_, *D*_c_, and *D*_2_—required for efficient waveguiding and optimized photon extraction efficiency into numerical aperture (N.A.) in the experimentally available collection optics of the μ-PL setup, i.e., N.A. = 0.4. The resulting target values provide a benchmark for subsequent growth optimization.

First, in order to find the preliminary value of the cuboid lateral side length at the QD height (*D*_c_) providing optimal confinement for single-mode propagation along the NW, the InP NW has been modeled as an infinitely long, square-based, untapered NW with a side length *D*_c_, as explained in the Experimental Section. In our case, the fundamental mode can be classified as degenerated HE_11_-like mode confirmed by the same effective refractive index of the *x* and *y* modes with a similar value to the circular cross-section case with the same cross-section area.^40^ Figure 2a shows the obtained normalized spontaneous emission rates as a function of *D*_c_. Consistent with literature reports for the NW-QD structures emitting at shorter wavelengths,^41^ the best confinement of the fundamental mode and the lowest off-axis losses are achieved for the ratio 0.22 < *D*_c_/λ < 0.26, with λ being the emission wavelength. For a smaller *D*_c_, the guided mode is not well confined within the NW, strongly leaking out, and as a result, a strong increase in the radiation losses γ is observed. In the case of a larger *D*_c_, the confinement gets weaker and after exceeding the cutoff value the structure becomes multimodal, so that the coupling into the fundamental mode β decreases. Because of different spatial distributions of the electromagnetic field for the different modes, e.g., with the field maximum near the NW edge, the radiation loss γ increases. In the next step, more realistic NW geometry was considered (experimentally obtained as shown in Figure 1e) to fine-tune the design and to identify the geometrical parameters crucial for achieving maximal photon EE from the structure (see the “3D model” in the Experimental Section). In the model, the bottom cuboid part where the QD is located has an *L*_c_ of 1 μm and a *D*_c_ of 330 nm, which is the value fine-tuned compared to the results obtained from the calculations of infinitely long NW. The second segment has a length *L*_2_ of 620 nm, while the third segment was set to *L*_3_ = 350 nm, with a top side length *D*_NP_ of 50 nm. The dipole (imitating the QD emitter) is placed at the bottom cuboidal part, and its position is optimized to be in the electric field maximum of the waveguide mode (Figure 2e). The obtained corresponding Purcell factor is close to 1, as seen in Figure 2a. The second segment is responsible for efficient light outcoupling. Increasing its length *L*_2_ causes a significant increase in directionality of emission translating into an increase in light extraction efficiency, as presented in Figure 2c. While a longer *L*_2_ value would be highly beneficial for the extraction efficiency (EE), there is a constraint between the optimal *D*_c_ and the maximum *L*_2_ that can be obtained. It can be seen that in the bottom NW part *D*_c_/λ = 0.22, so in the lower limit of *D*_c_ it was calculated as optimal. For the cost of slightly worse confinement, the second segment of the NW taper angle decreased while keeping the *D*_2_ small enough to minimize scattering from the top facet, which enhanced extraction efficiency within small N.A. Too small a *D*_2_ value on the other hand would increase the taper angle and block the optical mode to be efficiently outcoupled from the tip, which is shown in Figure 2d. For the experimental numerical aperture N.A. of 0.4, the change of EE with *D*_2_ is in the single percentage range, when *D*_2_ is varied in a broad range of 100–300 nm with a maximum at 250 nm. With increasing *D*_2_ value, the taper angle becomes smaller, so the optical mode is more efficiently guided to the NW tip for outcoupling. When *D*_2_ exceeds 250 nm, the NW tip volume becomes big, and reflection and scattering from the top interface start to be more significant and decrease the amount of the collected signal. The third segment of the NW is solely a consequence of the presence of gold droplets, and minimizing its size is beneficial since the electric field does not enter this part, as presented in Figure 2e. Calculated EE as a function of N.A. for the NW with fully optimized geometry (optimized within parameters reachable by the growth technology) is presented in Figure 2f. Overall, 46% photon EE was achieved for very high numerical apertures (for N.A. = 0.4 of the detection optics used in our experiments, the value is reduced to 15%). The Purcell factor shown in Figure 2g is slightly less than 1 due to the imperfect *D*_c_ value. The dependence can be traced back to the Purcell factor dependence on the group refractive index and mode confinement, which are affected by the NW diameter. This is the reason for the NW/substrate interface to reflect some of the light due to the difference between the effective refractive index value of the NW and the refractive index of the substrate, even though they are from the same material (modal reflectivity). This influences the optimal QD placement. The EE remains high for a certain range of emission wavelengths, as shown in Figure 2h. Spectrally broad extraction function is practically beneficial, as it does not require additional fine-tuning of the QD emission (redesign of the NW geometry) as long as it falls into the high extraction range. Thus, based on the calculations, the optimal values for the waveguide parameters in the emission wavelength range of our QDs result to be *D*_c_ = 330 nm, *L*_c_ > 450 nm, mainly to provide a uniform QD environment of the shell, *L*_2_ = 620, *D*_2_ = 250, and *L*_3_ = 350 nm or smaller. The QD must be placed at 450 nm from the NW base in order to minimize spreading of the QD emission (increase directionality of the emission) and further to be in the maximum of the optical mode electromagnetic field for the NW waveguide (Figure 2b).

**Figure 2:**
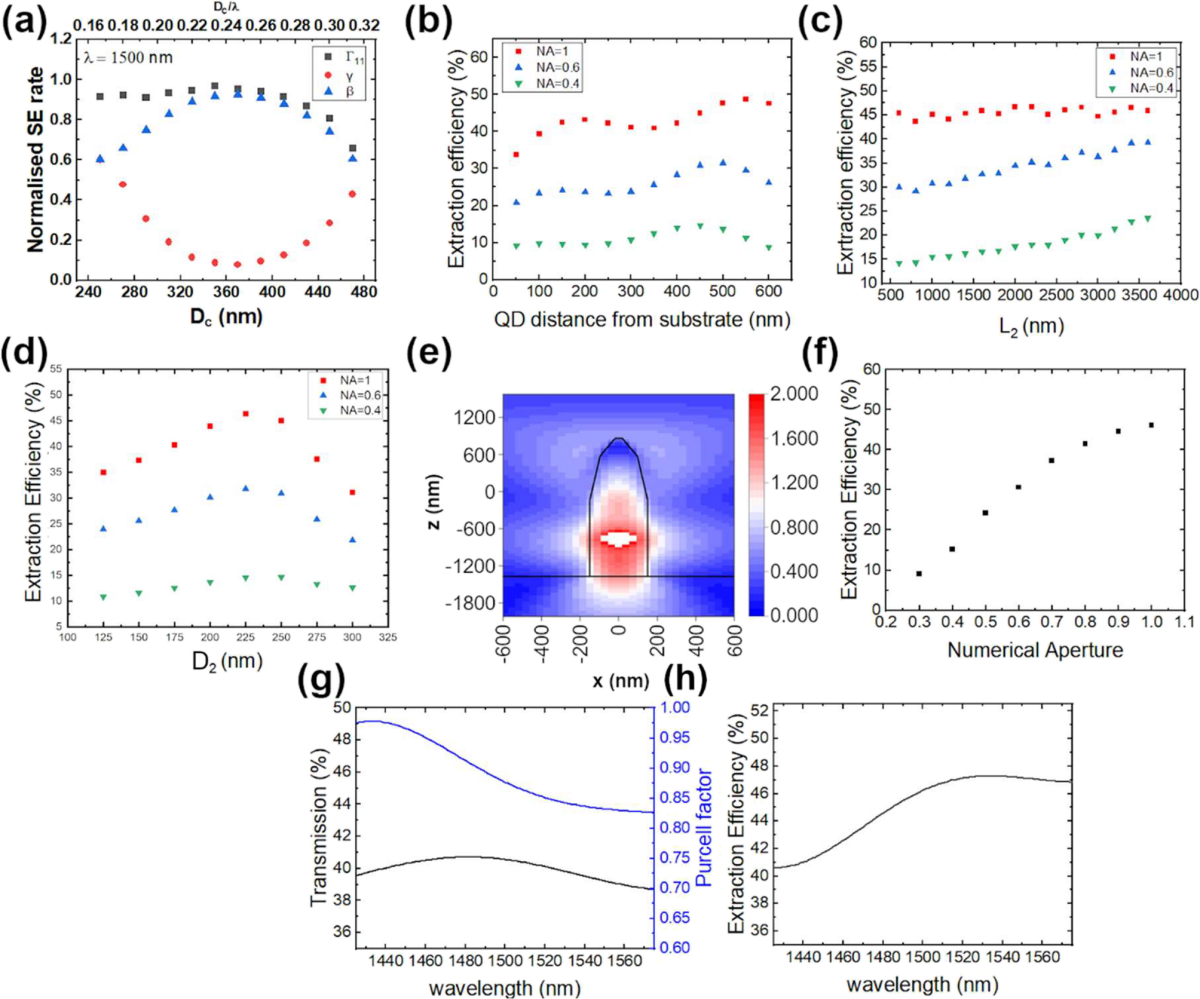
(a) Calculated normalized spontaneous emission rates of a radial dipole embedded on axis in an infinitely long, square-based InP nanowire. Emission rate into fundamental waveguide mode Γ_11_ (black squares), radiation losses γ (red dots), and coupling β factor (blue triangles) are presented as a function of NW side length *D*_c_ (bottom axis) and ratio between *D*_c_ and emission wavelength (top axis); (b) extraction efficiency as a function of QD position along the NW axis within different N.A. of collection optics for optimized tapered NW geometry; (c) extraction efficiency as a function of *L*_2_ within different N.A. of collection optics for optimized tapered NW geometry; (d) extraction efficiency as a function of *D*_2_ within different N.A. of collection optics for optimized tapered NW geometry; (e) electric field distribution (color coded) from dipole emission in a tapered NW (shape of the NW shown with a black solid line) on the InP substrate (marked with a horizontal black solid line) with optimized geometry parameters; (f) extraction efficiency as a function of N.A. of collection optics, for tapered NW with optimized geometry parameters; (g) transmitted power through a planar monitor above the optimized tapered NW (corresponding to N.A. of collection optics equal to 1)—black solid line (left vertical axis), and Purcell factor values (blue line, right vertical axis) for different dipole emission wavelengths; and (h) extraction efficiency as a function of the dipole emission wavelength in optimized tapered NW.

### Waveguide Growth Optimization

From the FDTD calculations, it is evident that the waveguiding effect is mainly determined by the value of *D*_c_, with the optimum found at *D*_c_ = 330 nm for the emission wavelength of λ = 1500 nm and by the position of the QD along the NW axis. While the latter can be easily controlled by controlling the growth time of the first InP segment prior to the QD growth, *D*_c_ is strongly linked with other morphological parameters of the waveguide. Furthermore, achieving high photon EE also requires proper control of the tapering in the second segment. To quantitatively compare the morphology of different samples, we introduce two parameters that capture the most relevant aspects of the NW morphology. The first one is the tapering parameter of the second segment, which deals mainly with the photon EE from the waveguide and is defined as

$$t_{2}=\frac{1}{2}\cdot \frac{D_{c}-D_{2}}{L_{2}}$$

This parameter is zero for an untapered segment, negative for divergent tapering, and positive for convergent tapering. Inserting the optimal values of *D*_c_, *D*_2_, and *L*_2_ obtained with the FDTD simulations leads to *t*_2_ optimal values in the range between 0.020 (for *L*_2_ = 2 μm) and 0.065 (for *L*_2_ = 620 nm), with smaller values being preferable. The second parameter is ϵ_*D*_c__, the relative percentage deviation of *D*_c_ from the target value of *D*_c_^target^ = 330 nm:

$$\epsilon_{D_{c}}=\frac{D_{c}-D_{c}^{\mathrm{target}}}{D_{c}^{\mathrm{target}}}\times 100$$

It provides a direct measure of how much the cuboidal segment lateral side length deviates from the optimal value to achieve the waveguiding effect, with ϵ_*D*_c__ = 0 when *D*_c_ is equal to the target value *D*_c_^target^ = 330 nm. Both parameters are measured for each sample, as discussed in Section S1 of the Supporting Information (SI). In the following, we focus on the growth of the InP shell using the previously described NW core as a template. Since the NW morphology is closely linked with the diffusion length of In adatoms on the sidewalls, we investigate the dependence of the InP shell-relevant quantities on the growth parameters that could affect it: growth temperature, V/III ratio, and total TMIn flux. Results on the temperature dependence are presented in Section S2 of the SI, showing that temperature variations destabilize the catalyst, resulting in nonstraight NWs. Here, we focus on the effects of the V/III ratio and the TMIn flux. Figures 3a–d show 45°-tilted SEM images of representative NWs with an InP shell grown at constant TMIn line pressure (0.4 Torr) and varying TBP line pressure. The resulting V/III ratios are 2, 3, 4, and 5, respectively. All samples are grown with similar average NW total lengths to exclude length-dependent effects. Panel (e) reports the dependence of *t*_2_, ϵ_*D*_c__, and *L*_3_ on the V/III ratio. In this case, no strong dependence is observed, with *t*_2_ remaining close to the optimal range (in green in the graphic), while *D*_c_ deviates by ∼20% below the optimal value for all the V/III ratios explored. Panel (f) reports the InP shell axial and radial growth rates, calculated at the QD height, which appear unaffected by the V/III ratio. This indicates that, in our growth regime, the process is limited mainly by the TMIn transport rather than group V supply. Also, the cuboid cross-section of the NWs preserves a square shape bounded by {110} facets, with only minimal growth of {100} facets, equivalent across all the V/III ratios explored. A typical cross section observed for this series is reported in Figure S3 of SI. This behavior differs from that reported in ref 42, which shows {100}-faceted NWs dependent on the V/III ratio, but agrees with ref 43, suggesting that our samples are grown under different flux conditions compared to the first work.

**Figure 3:**
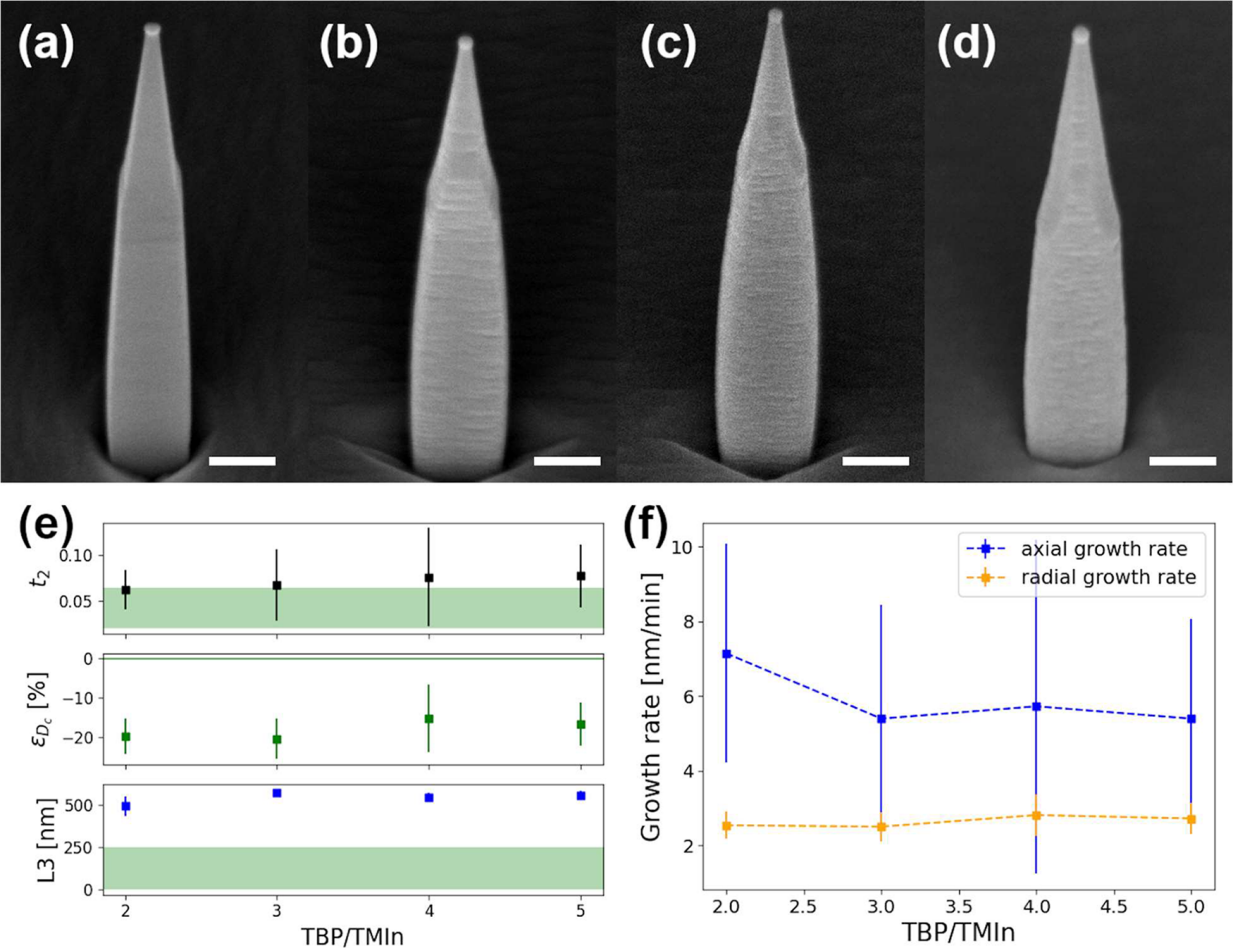
45°-tilted view SEM images of the samples grown with a ratio of the TBP and TMIn line pressures of: (a) 2; (b) 3; (c) 4; and (d) 5. Scale bar in panels (a–d) is 200 nm; (e) dependence of the average *t*_2_, ϵ_*D*_c__, and *L*_3_ by the V/III ratio. In green, the optimal value range for each quantity; and (f) axial (blue) and radial (orange) growth rate dependence by the V/III ratio.

Since the V/III ratio is not influencing the NW morphology, we chose to keep it at 4.0 and to investigate the effect of the TMIn line pressure.

Figure 4a–e show 45°-tilted side-view SEM images of NWs with an InP shell grown at TMIn line pressures ranging from 0.2 to 0.6 Torr. These five samples have a similar average NW total length of (1400 ± 100) nm. The NW morphology shows a clear dependence on the TMIn line pressure. Increasing the TMIn line pressure strongly modifies the relative lengths of segments *L*_3_ and *L*_2_, with the second segment disappearing completely at the highest TMIn value (panel e). Additionally, microfaceting of the sidewalls becomes visible at higher TMIn fluxes, in agreement with ref 42. This effect has been attributed to a reduction in the diffusion length of In adatoms under higher fluxes,^44,45^ leading to their incorporation closer to the impinging site and the formation of higher-energy microfacets. The sidewall surface roughness related to microfaceting is negligibly small compared to the emission wavelength and short optical path at which scattering could occur for light propagating along the NW, so it will not influence scattering loss significantly. Panel (f) shows the relevant waveguide parameter changes between the samples. *t*_2_ shows a nonmonotonic trend, with the most favorable value obtained at TMIn = 0.4 Torr. At the highest TMIn line pressure (panel (e), a single tapered segment forms above the cuboidal base, eliminating the distinction of the second segment, despite *D*_c_ being close to the target value for the waveguiding effect. *L*_3_ increases with the TMIn line pressure, making lower TMIn line pressures preferable. The lower value of ϵ_*D*_c__ observed at TMIn = 0.4 Torr compared to 0.3 Torr is likely due to small temperature fluctuations naturally occurring from sample to sample in the CBE system; these fluctuations do not significantly affect the overall trends discussed in this work. Figure 4g compares the dependence of the average total axial growth rate (blue curve) and the average radial growth rate at the QD height (orange curve) on the TMIn line pressure. The average over many NWs (at least 20) is considered since in the same as-grown sample, there are always differences in morphology from NW to NW, usually arising from small temperature inhomogeneities or variations of the actual precursor fluxes because of the different shadowing experienced by neighboring NWs due to their random distribution on the substrate. However, these fluctuations do not affect the average quantities, and all samples remain comparable within the experimental uncertainties. Employing in Figure 4g a linear fit as a first approximation, it is possible to extrapolate what is the change of each growth rate by the varying TMIn line pressure, obtaining that by decreasing TMIn by 0.1 Torr, the axial growth rate decreases approximately 4 times compared to the radial growth rate. From these results, the best InP shell growth condition is obtained at TMIn = 0.4 Torr, giving the best value for *t*_2_ = 0.09 ± 0.01 and ϵ_*D*_c__ = −0.16 ± 0.04, below the target value. We will refer to this best sample in the following as the “template” sample.

**Figure 4:**
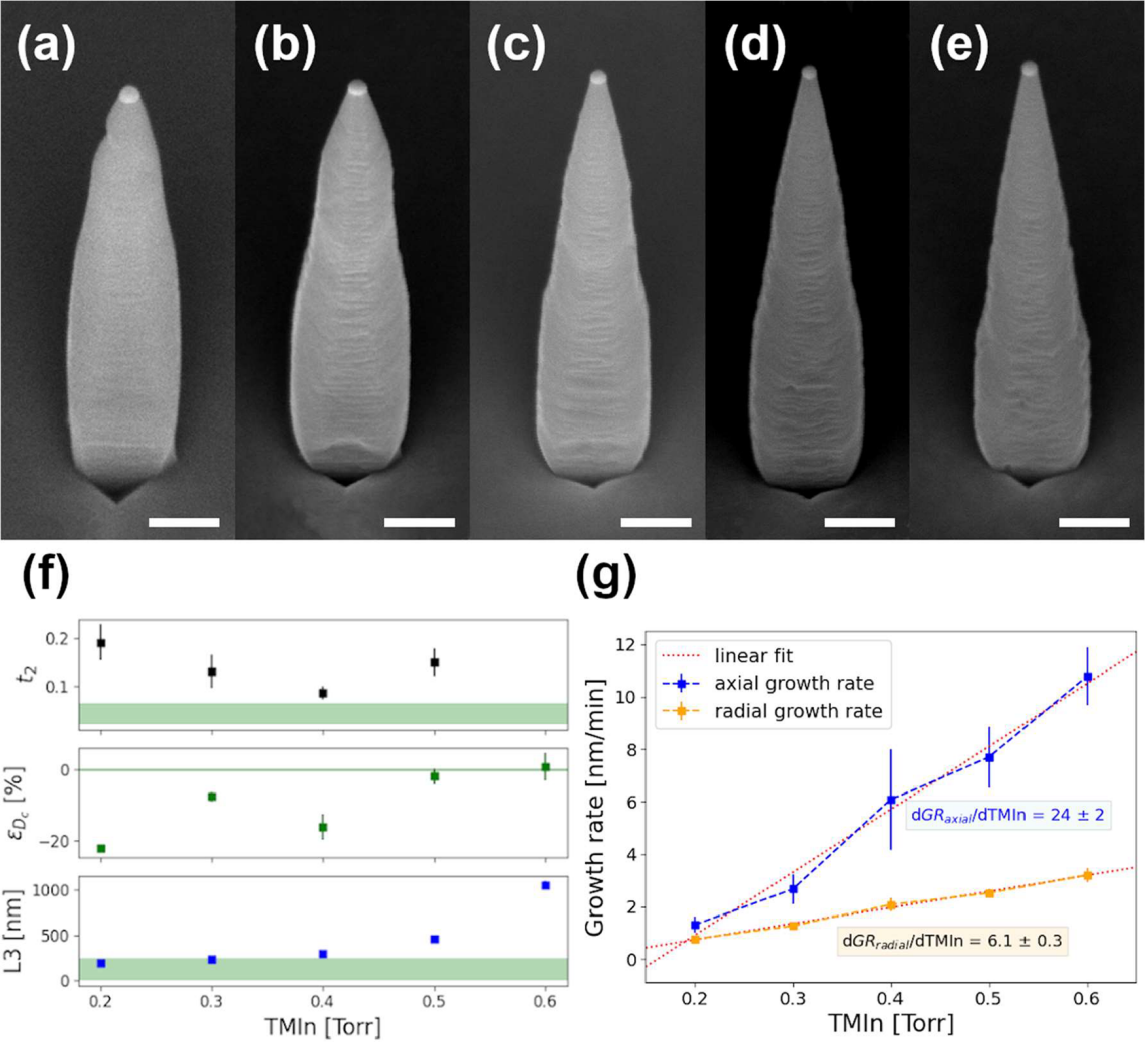
45°-tilted side view SEM images of representative NWs grown by keeping V/III = 4 and employing TMIn line pressure for the shell: (a) 0.2 Torr; (b) 0.3 Torr; (c) 0.4 Torr; (d) 0.5 Torr; and (e) 0.6 Torr. In all the images, the scale bar corresponds to 200 nm, and the NW length is on average (1400 ± 100) nm for all the samples; (f) dependence of the average *t*_2_, ϵ_*D*_c__, and *L*_3_ on the TMIn line pressure employed for the shell growth. In green, the optimal value range for each quantity is highlighted; (g) average axial NW growth rate (in blue) vs average radial growth rate at the QD height (in orange) dependences on the TMIn line pressure employed for the shell growth. Red dotted lines are the linear fits, with which the variations $\frac{{\mathrm{dGR}}_{\mathrm{axial}}}{d\mathrm{TMIn}}$ and $\frac{{\mathrm{dGR}}_{\mathrm{radial}}}{d\mathrm{TMIn}}$ of the growth rates with TMIn have been computed.

To further optimize its morphology, *D*_*c*_ must be increased toward its optimal value while keeping *t*_2_ close to the optimal range. Two strategies were tested, as schematized in Figure 5a.

**Figure 5:**
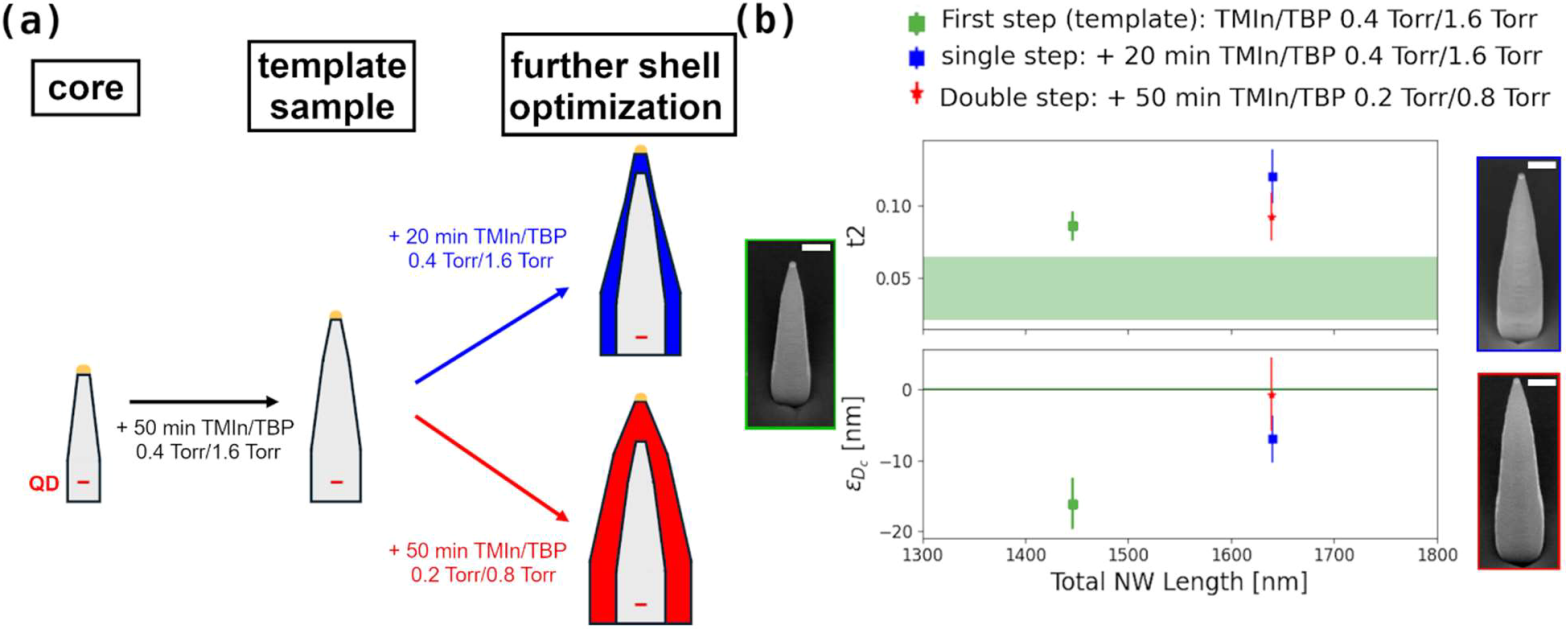
(a) A schematic of the different growth approaches; (b) comparison of the waveguide relevant quantities between the template sample (green points) with a sample grown in the same conditions but for longer time (blue points) and a sample grown with the double step approach explained in the main text (red points). A SEM image of the three samples is also reported in the green, blue, and red insets, respectively (scale bar is 200 nm).

In the first approach (blue arrow), starting from the template sample, the growth is simply extended under the same line pressures (i.e., TMIn/TBP = 0.4/1.6 Torr) for an additional 20 min, yielding a total NW length of (1640 ± 30) nm. Figure 5b compares the relevant waveguide parameters of this longer sample (blue points) with those of the template sample (green points), with 45°-tilted side-view SEM images of a representative NW reported in the blue and green insets, respectively. While ϵ_*D*_c__ moves closer to its optimal value, *t*_2_ increases a lot beyond the upper limit of the target range. The second approach (red arrow in Figure 5a) consists of a two-step InP shell growth. After the growth of the template sample, the line pressures are lowered to TMIn/TBP = 0.2/0.8 Torr for an additional 50 min growth. The choice of lowering TMIn during the second step growth is motivated by the need to promote radial growth, while limiting further axial elongation, as suggested by the growth rate comparison in Figure 4g. The measured InP shell parameters for this sample are shown in Figure 5b (red points) as well as a representative NW SEM image in the red inset. Compared to the extended-growth strategy (blue spots), the double-step growth approach successfully tunes ϵ_*D*_c__ to the optimal value ϵ_*D*_c__ = 0 while keeping *t*_2_ closer to the target range. It must be mentioned that *t*_2_ is 0.09 ± 0.02, thus slightly above the optimal value. While *D*_c_ = (328 ± 17) nm for this sample is in the optimal range and also *L*_2_ = (628 ± 58) nm is the value required from the calculations, the main affecting parameter for the higher value of *t*_2_ is *D*_2_ = (212 ± 7) nm, slightly lower than the optimal value. Overall, these results demonstrate that the double-step strategy improves the InP shell morphology and also highlights the potential for further refinement. In particular, we believe that carefully tuning the relative durations of the two shell growth steps should allow us to balance the axial and radial contributions more precisely, ultimately converging toward a fully optimized morphology for efficient light guiding.

### Optical Characterization

After optimizing the InP shell morphology, we investigated the optical properties of the grown samples using μ-PL measurements at 10 K. In particular, we studied samples that differ in their key waveguide parameters in order to observe how the emission intensity depends on the NW geometry. Figure 6a–d show measured μ-PL spectra as a function of excitation power for four different samples. Each panel shows the spectrum from a single QD-NW. The NW spatial density on the investigated samples was low enough (10^–2^ NWs/μm^2^) compared to the laser spot size (single micrometer range) to avoid exciting multiple structures at once. Panel (a) shows the spectra from bare NW cores (those previously shown in Figure 1d), which have a small cuboid side length *D*_*c*_ = (163 ± 15) nm (i.e., ϵ_*D*_c__ = −0.50 ± 0.05). Panel (b) is measured on the template sample, grown with a single step of InP shell under TMIn and TBP of 0.4 and 1.6 Torr, respectively, and corresponding to the green points of Figure 5b. As already discussed, this sample is characterized by a smaller *D*_c_ = (277 ± 17) nm compared to the optimal value (i.e., ϵ_*D*_c__ = −0.16 ± 0.04) and *t*_2_ = 0.09 ± 0.01. Panel (c) concerns a sample grown under the same conditions of the template sample, but for longer time (95 min) in order to have a *D*_c_ > *D*_c_^target^. This sample has *D*_c_ = (369 ± 9) nm (i.e., ϵ_*D*_c__ = 0.12 ± 0.03) and *t*_2_ = (0.10 ± 0.02). Finally, Figure 6d presents the spectra measured on the sample with the optimized InP shell (red points in Figure 5b). This sample has *D*_c_ = (328 ± 17) nm (i.e., ϵ_*D*_c__ = 0.01 ± 0.05), *t*_2_ = (0.09 ± 0.02), values that are closest to the target design. All of the spectra display multiple emission lines, confirming 3D spatial confinement of the carriers within QD potential. Depending on the details of QD height and As content, the emission from these samples can be observed in the range of 1.35–1.55 μm, reaching the telecommunication C-band (see, e.g., Figure 6c). The emission consists of many lines resulting from emission from various excitonic complexes in their ground and possibly also excited states. This is related to the fact that the investigated dots are large for emission in the third telecom window, resulting in a dense ladder of confined states corresponding to many closely spaced emission lines merging into emission bands. Nonresonant excitation without specific polarization and charge control allows for formation of various complexes of both signs. Identification of the origin of the emission lines is beyond the scope of this work, as it is not necessary for the sake of our analysis, which focuses on the influence of the NW geometry on the emission intensity that can be extracted from the structures. The differences in emission energy between the samples reflect small unintentional variations between QD morphologies, mostly slightly different QD heights that naturally occur from sample to sample during the growth and are also present in the same sample (with an average QD height of (10 ± 2) nm, as previously mentioned). The full width at half-maximum for observed emission lines is on the order of a few meV, which can be attributed to the spectral diffusion due to charge fluctuations around the QD (this parameter has not been optimized in this work). Coulomb interaction with time-varying charge environment causes spectral shifts of emission lines, which are averaged by the detector as they are faster than the integration time for a single spectrum (tens of seconds). This leads to inhomogeneous broadening observed in the experiment which depends on the amplitude of above-mentioned fluctuations, as already discussed in our previous work.^30^ The increased background level at shorter wavelengths is caused by a broad signal centered at 1150 nm, coming from a thin 2D heterostructure of the QD material and InP arising during growth and covering the whole substrate, around the NWs (see Figure S4 in the SI). To compare the different NW geometries realized experimentally in terms of photon extraction efficiency, we use the integrated emission intensity from a single NW-QD as a figure of merit. This corresponds to summing up all photons emitted by one QD independently of the exact emitting state. The integrated emission intensity is obtained by numerically integrating the spectrum in the whole spectral range of NW-QD emission from 1350 to 1550 nm. The spectral range for signal integration was chosen in such a way to minimize the influence of the 2D-related signal on the integrated emission coming from the QD-NWs. The NW-to-NW differences are accounted for by measuring multiple NW-QD structures in each sample and averaging the results. This will not give the absolute value of the photon EE, but it will reflect well the relative photon EE of the different designs. The underlying assumption is that QDs in different samples have approximately the same internal quantum efficiency, supported by the fact that the QD parts of all of the structures have the same nominal parameters and growth conditions. An even stronger requirement is typically used in the case of the determination of photon extraction efficiency, where 100% internal quantum efficiency of the emitter is assumed. This is not needed in our case. In the case of continuous wave measurements, we assume a similar PL decay lifetime. This is justified as it has been verified experimentally to be relatively insensitive to the details of QDs size and composition in the parameter range relevant for the investigated samples (see Section S5 in the SI) and also the exact design of the NW waveguide. This suggests that the Purcell effect on spontaneous emission lifetime is counteracted by nonradiative recombination due to proximity of NW sidewalls and QD in small diameter NWs. Additionally, the μ-PL setup used (see Experimental Section) is less sensitive to the differences in NW-to-NW adjustment than the setup with fiber-coupled single-photon detectors, which would have been used for photon EE measurements. Due to their relative character, the provided results are also independent of the transmission of the experimental setup, which is another source of uncertainty in the case of evaluation of absolute photon EE. Figure 6e presents the excitation power dependence of the integrated emission intensity. Error bars show statistical error for 3 randomly selected NWs from each sample. It is clearly seen that NWs with optimized geometry parameters (in red) show the highest integrated intensity at saturation power, almost 1 order of magnitude higher than the core sample, which proves the validity of the numerical calculations and successful realization of the proposed designs in growth. However, it is difficult to directly compare the emission intensity enhancement to the increase in the calculated EE. This is mainly because the calculations do not include nonradiative recombination and because the quantitative comparison of theoretical results works well only if the guided mode is confined in the NW, and as a result, the Purcell factor is in the range of 1. For a NW sample with *D*_c_ = 277 nm, being in the regime of sizes where this condition is satisfied, we calculated that the Purcell factor decreased by a factor of 3. Additionally, from Figure 2a, it can be seen that a decrease in NW diameter by 100 nm from the optimal value results in an increase in radiative losses from 5 to 50%, due to poor light confinement. If we combine it with a few percentage points of EE that can be lost due to unoptimized taper engineering and QD placement, we can explain an almost 10-fold decrease in the predicted EE. The lower signal level from the sample with a too thick shell is most likely a consequence of the worse (in view of EE) taper angle. NWs from all the samples have similar total height. By increasing diameter *D*_c_ and keeping the *D*_2_ value constant, the taper angle increases, which lowers the amount of the collected signal within a given numerical aperture. It is also worth noticing that similarly to ref 18, we observed a decrease in QD emission intensity with increasing emission wavelength for samples with the same shell thickness. This additionally supports the dominant effect of the shell thickness on the observed QD emission intensity. The NW geometry was optimized for 1500 nm corresponding to the ground-state emission and most interesting in view of applications (III telecom window). Even though QD-NWs emit in a broader spectral range that was subject to numerical optimization, the design works well, and the integrated intensity does not have to be rescaled. This is because enhancement of the EE in the NWs is a spectrally broad effect with EE changing only by 7 percentage points over the whole spectral range of QD emission. It is possible to further red-shift the central emission wavelength to 1550 nm by adjusting the QD composition (increasing the As content will shrink the band gap and make the confinement potential deeper) or by increasing the QD size exploiting the quantum size effect (the most efficient will be an increase in the QD height). The efficiency of both tuning knobs has been demonstrated in our previous work at shorter wavelengths.^30^

**Figure 6:**
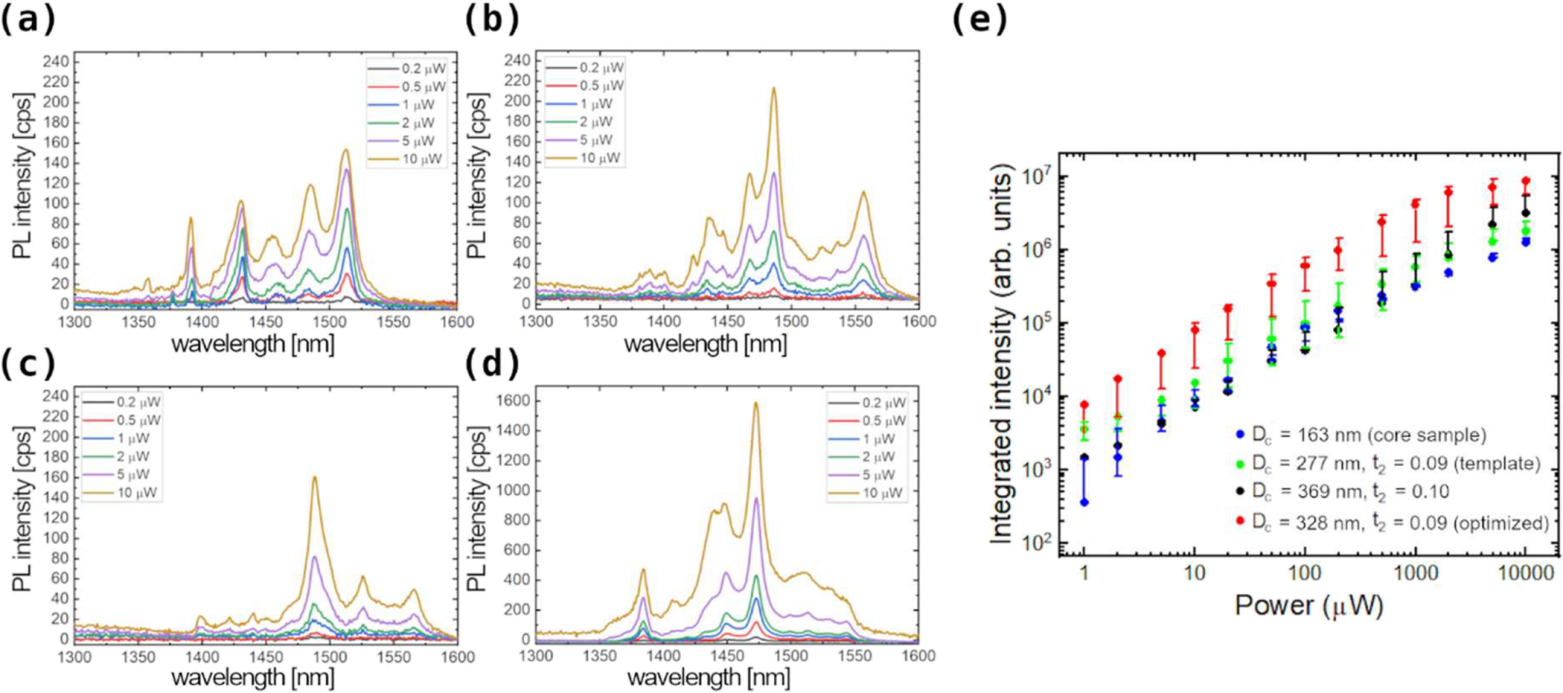
μ-PL spectra measured at *T* = 10 K with different excitation powers using 640 nm nonresonant excitation of samples with (a) *D*_c_ = (163 ± 15) nm, being the core sample; (b) *D*_c_ = (277 ± 17) nm and *t*_2_ = 0.09 ± 0.01, being the template sample; (c) *D*_c_ = (369 ± 9) nm and *t*_2_ = 0.10 ± 0.02; and (d) *D*_c_ = (328 ± 17) nm and *t*_2_ = 0.09 ± 0.02. This is the sample with the optimized InP shell; (e) integrated intensity of emission as a function of excitation power for the NW-QDs of panels (a–d). Error bars mark the statistical error for measurements on three arbitrary NWs from each sample.

## Conclusions

In summary, we have demonstrated the controlled growth of InP waveguides around ZB InAs_*x*_P_1–*x*_ QDs without any pregrowth substrate fabrication or the use of SAE-VLS approaches commonly adopted in literature. By finely tuning the growth parameters to balance the axial and radial contributions, we realized optimized shell morphologies that function as efficient waveguides, integrated directly during growth. The typical morphology observed in these ZB NWs is characterized by a three-segment morphology. The investigation of the dependence of the InP shell morphology on the growth conditions allowed us to determine the role of each growth parameter and the tunability range of the waveguide parameters. FDTD simulations guided the design of the optimal geometry, and the experimental realization confirmed the predicted enhancement of the photon extraction. Furthermore, we report for the first time to our knowledge emission in the telecom C-band from defect-free ZB InAs_*x*_P_1–*x*_ QDs embedded in NWs with in situ grown InP waveguides. The optimized structures exhibit nearly an order of magnitude higher emission intensity compared with unoptimized designs, underlying the effectiveness of our approach. These findings establish ZB InAs_*x*_P_1–*x*_ NW-QDs as a versatile platform for telecom-band light sources, combining high crystal quality, spectral tunability, and enhanced light extraction.

## Supplementary Material



## Notes

Additional experimental details, materials, and methods; NW diameter at different NW heights along the axis of NWs; 45°-tilted side view SEM images of samples with the InP shell grown at a different growth temperature; top view SEM image of the typical InP NW cross section; low-temperature (10 K) high excitation photoluminescence spectrum (13 mW) of a reference sample without NWs; and low-temperature (10 K) PL spectrum and dispersion of PL decay time measured on the ensemble of NW-QDs at a low excitation power of 25 μWs (PDF)

## Abbreviations

NW: nanowire
NW-QD: nanowire quantum dot
QD: quantum dot
μ-PL: microphotoluminescence
TMIn: trimethylindium
TBP: *tert*-butyl phosphine
TBAs: *tert*-butyl arsine
CBE: chemical beam epitaxy
VLS: vapor–liquid–solid
SAE: selective area epitaxy
SEM: scanning electron microscopy
