# Supplementary material for: Enhanced Photon Extraction through Optimized Waveguide Geometry for Zincblende InAsP/InP Nanowire Quantum Dots Emitting in the Telecom Range
Source: ACS Appl Nano Mater. 2026 Jan 14;9(4):1837–48. doi: 10.1021/acsanm.5c04842 (PMC12865759; doi:10.1021/acsanm.5c04842)
Supplement: Supplementary file 1 [file an5c04842_si_001.pdf]

Supporting Information

Enhanced Photon Extraction through Optimized Waveguide Geometry for Zincblende InAsP/InP Nanowire Quantum Dots Emitting in Telecom Range

Giada Bucci[1], Tomasz Gzyl[2], Anna Musiał[2], Valentina Zannier[1], Fabio Beltram[1], Wojciech Rudno-Rudziński[2], Grzegorz Sęk[2] and Lucia Sorba[1]*

[1] NEST Scuola Normale Superiore and Istituto Nanoscienze CNR, 56127, Pisa, Italy

[2] Department of Experimental Physics, Wrocław University of Science and Technology, 50-370, Wrocław, Poland

*Corresponding Author: lucia.sorba@nano.cnr.it

S1 – Waveguide parameters measurements

As discussed in the main text, the nanowires (NWs) in this study typically exhibit a three-segment morphology. To understand how the waveguide parameters depend on the growth conditions, we extracted the characteristic dimensions defined in Figure 1(d) of the main text: L_c, D_c, L_2, D_2 and L_3. These quantities are obtained from 45°-tilted SEM images, analyzed using imageJ software [1]. From each NW SEM image, the diameter is measured at regular intervals of about 10 nm along the NW axis, starting from the catalyst nanoparticle and moving towards the NW base. At each measurement point, we recorded both the NW diameter and its distance from the catalyst, thereby obtaining a diameter profile along the entire NW length. Examples of such profiles are reported in Figures S1(a-d), together with the corresponding SEM images from which the profile is measured. These examples are taken from the same samples shown in Figure 1 of the main text and clearly illustrate how the NW morphology changes with increasing total NW length. Figure S1(d) shows the typical three segment structure observed in all our samples, where each region is characterized by a distinct slope. The transition points between the three segments were identified (and highlighted in red in the graph), allowing us to determine L_c, L_2 and L_3, together with D_c and D_2. This procedure is applied systematically to at least 15 NWs for each sample, and the average values and their standard deviations are then calculated. From these quantities, we further derived the parameters t_2 and ϵ_{D_c}, according to the definitions introduced in the main text.

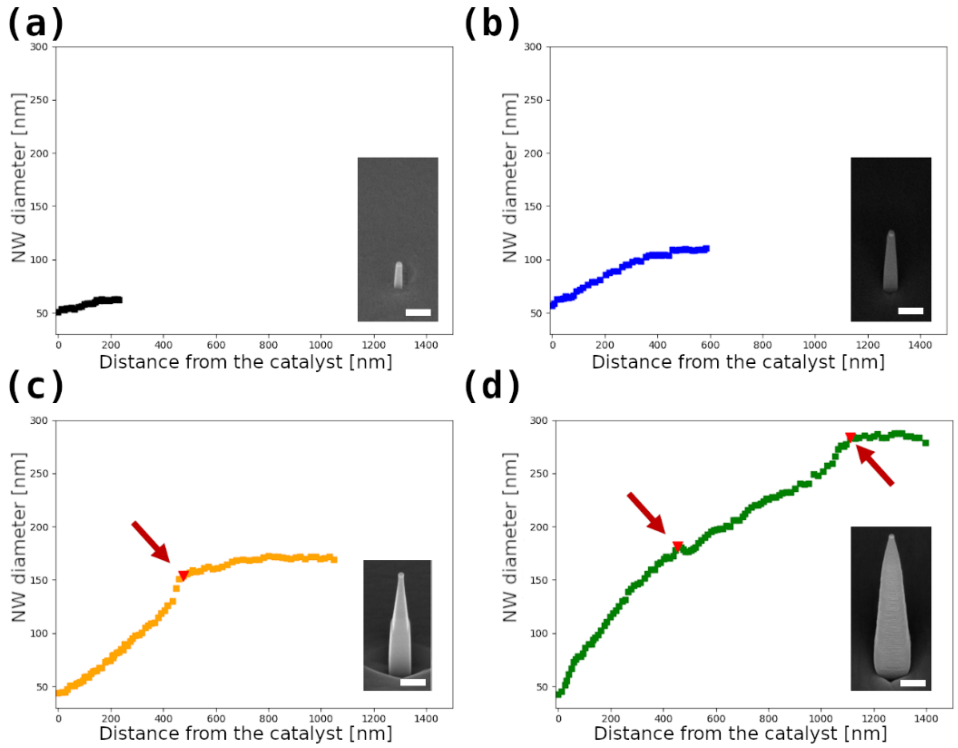

Figure S1. NW diameter at different NW height along the axis of NWs with total length: (a) (290 ± 60) nm; (b) (690 ± 130) nm; (c) (1140 ± 20) nm; and (d) (1446 ± 94) nm. In red and with red arrows are highlighted the points in which a change of slope is observed and employed for the estimations of the relevant quantities

S2 – Change in the InP shell growth with temperature

The growth temperature is surely an important growth parameter that acts on both the radial growth of the NW and the axial one, thanks to changing of the group III adatom diffusion. In particular, it has been observed in literature that a decrease in temperature typically leads to an enhancement of the radial growth rate of the NW[2], while higher temperature could lead to thermal decomposition of the NW facets in zincblende InP NWs grown along the <100> direction, with a change in the cross-sectional shape of the NW. Since we want to avoid such thermal decomposition

and we want to enhance the radial growth in order to grow a waveguide, we decided to grow the InP shell at two different temperatures lower than the temperature employed for the QD growth. Figure S2 presents two different samples grown with an InP shell of 50 minutes using TMIn and TBP line pressures of 0.4 Torr and 1.6 Torr, respectively, at different temperatures. In particular, panel (a) shows a sample in which the temperature is decreased by 20°C after the growth of the core with 5 minutes-long linear ramp, while linearly ramping TMIn and TBP line pressures from the ones of the core to the ones of the shell. In this case, the clear effect is a destabilization of the catalyst nanoparticle, leading to a not straight growth. If we decrease the shell temperature by 10°C, as in the sample of panel (b), kinking of the NW is observed, leading again to not straight growth. This behavior suggests that the growth temperature window for our NWs is quite narrow and the growth temperature of the shell must be kept constant and equal to the growth temperature of the NW core.

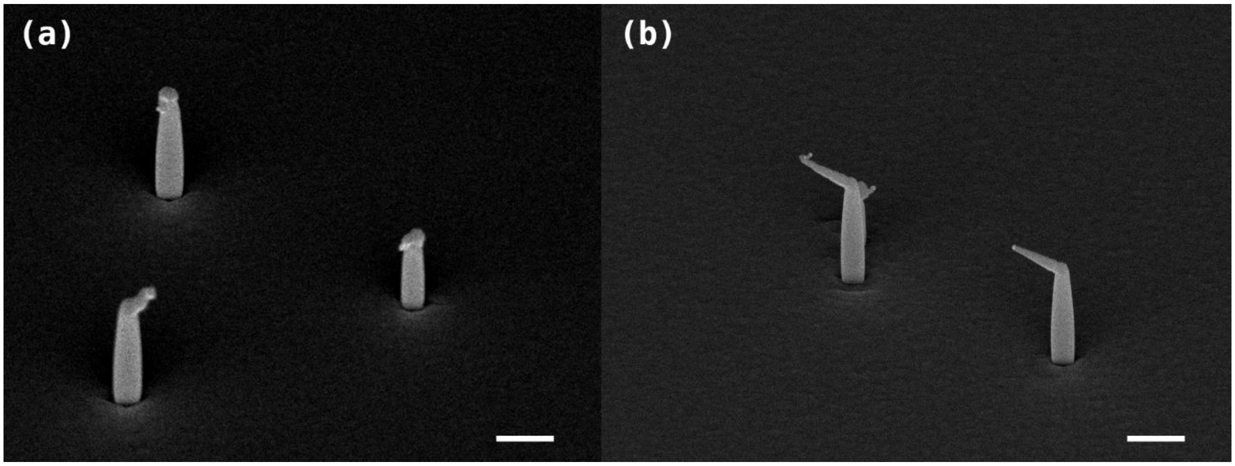

Figure S2. 45°-tilted side view SEM images of samples with the InP shell grown at a temperature (a) lower than 20°C compared to the QD growth, and (b) lower than 10°C compared to the QD growth. In both images, the scale bare is 500 nm.

S3 – NW typical cross section at TMIn 0.4 Torr

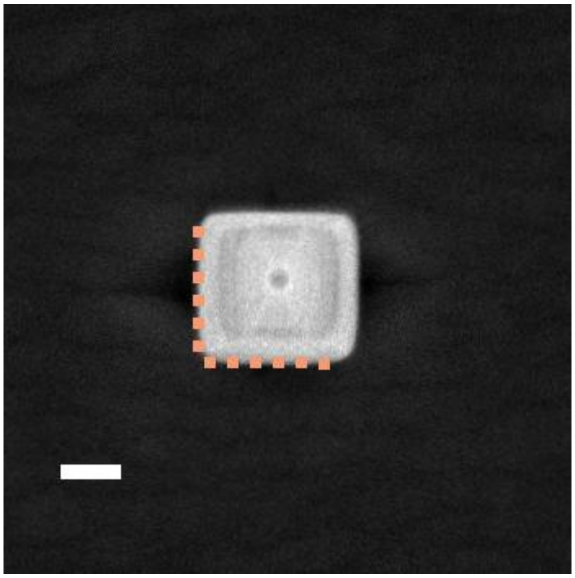

Figure S3. Top view SEM image of the typical InP NW cross section observed. The {110} facets of the cuboidal part of the NW are highlighted with orange dots in the image and are identified in reference to the cleavage plane of the InP(100) wafer. Scale bar is 100 nm.

S4 – Photoluminescence spectrum of the 2D heterostructure accompanying QD-NW growth

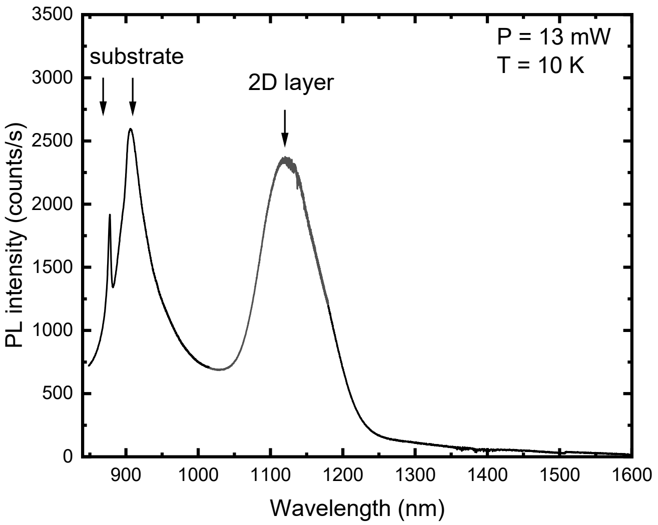

Figure S4. Low-temperature (10 K) high excitation photoluminescence spectrum (13 mW) of a reference sample grown using the same recipe as samples described in the main manuscript, but without Au catalyst. In such conditions no QD-NW formation takes place, but a 2D growth of the same materials as in the target sample. Emission from 2D heterostructure of QD material and InP centred at 1150 nm and from InP substrate (InP bandgap and donor-acceptor transition occurring due to Fe doping[3]) are clearly visible in the spectrum.

S5 - Photoluminescence decay times

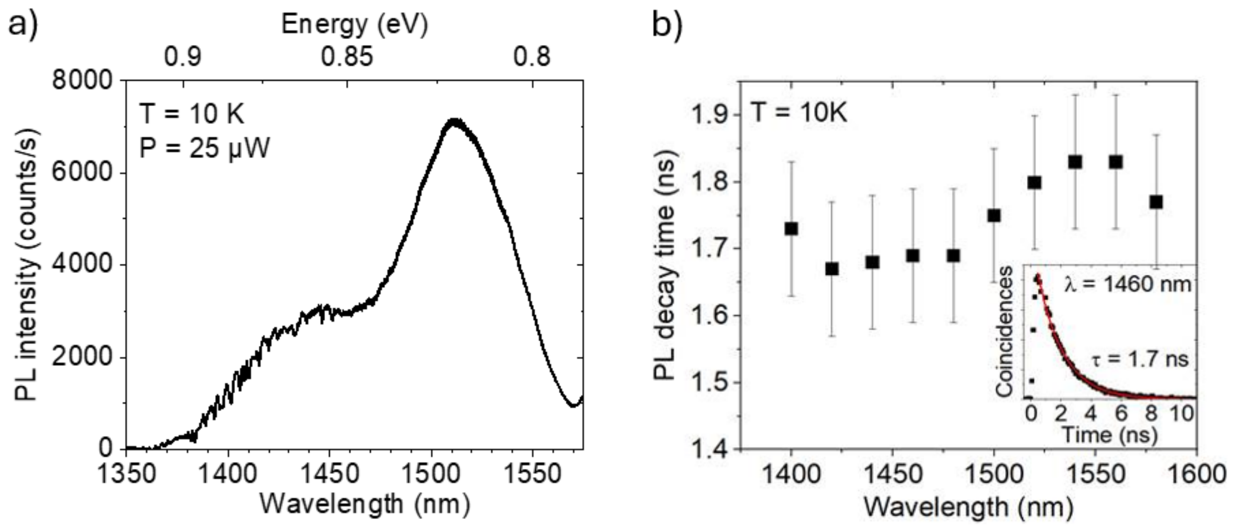

Figure S5. a) Low-temperature (10 K) photoluminescence spectrum measured on the ensemble of NW-QDs at low excitation power of 25 μW; b) dispersion of PL decay times measured in the same experimental conditions. Inset shows exemplary decay for 1460 nm wavelength (black data points) together with monoexponential fit to experimental data (red solid line).

To verify whether the photoluminescence (PL) decay times are similar for different NW-QDs, a series of measurements has been performed on a sample with 10 nm height and As content of (85 ± 2) %, to cover the spectral range of interest. The QD parameters in this sample are similar to the one discussed in the main text, however it is grown at slightly different conditions, with higher NWs density of (0.7 ± 0.3) NWs/μm^2 and with only a hundred-nm InP passivation shell around the QD, unoptimized with respect to photonic confinement and for the extraction efficiency. Sample with higher NW spatial density provided higher photoluminescence signal intensity which allowed performing time-resolved PL measurements. For this purpose, averaging over emission from many QDs is beneficial.

Investigated structures were cool down to 10 K in closed-cycle He refrigerator. The non-resonant excitation was provided by a pulsed semiconductor laser at 805 nm wavelength. It provides train

of ~50 ps long pulses with 80 MHz repetition rate. The excitation power has been kept in the low excitation regime (25 μW measured before the cryostat). The excitation beam was focused on the sample surface and signal was collected with lens with long focal length (150 mm), providing excitation spot diameter on the sample surface in a range of 100 μm. This ensures that many NW-QDs are excited at the same time, so the PL decay time dispersion is measured on the signal from NW-QDs ensemble. The signal was first spectrally filtered by 1 m focal length monochromator. The spectrally narrow portion of the signal (~0.65 nm) was further coupled to a standard single-mode telecommunication fiber connected to NbN semiconductor nanowire single photon detector for time-resolved measurements (quantum efficiency > 85%, dark count rate < 15 Hz and timing jitter < 40 ps). The trigger signal for the time tagging electronics is provided by Si diode using pick-off of the optical laser output. The measurements were performed in a time-correlated single photon counting mode with time-bin width of 100 ps. The central wavelength has been varied by changing the angle of 150 grooves/mm grating in the monochromator and a PL decay trace was measured every 10 nm over the PL band for a given sample. The corresponding PL spectrum was measured using an InGaAs multichannel array detector mounted on the second exit port of the monochromator to set up the spectral range and excitation power for the time-resolved measurements.

The results obtained are presented in Figure S5. Each of the time traces (for a given central wavelength) was fitted with monoexponential decay to extract the PL decay time. The overall accuracy of the method was determined based on the temporal characteristics of the experimental setup and the quality of the fits to be ± 100 ps. All determined PL decay times fall into the range of 1.67 to 1.83 ns. The observed variation of 0.16 ns (<10%) over the spectral range of 160 nm is relatively small and would not change the conclusions from comparing integrated PL intensity for

different NW geometries. In the latter case the differences between various designs are much larger than 10%, up to the level of one order of magnitude. Explaining the details of PL decay time dependence on emission wavelength is beyond the scope of the current manuscript and requires further investigation. It could result from the variation of QD composition and/or height within the QD- NW ensemble or due to the fact that different states are dominantly emitting in the short- and long-wavelength tail of the ensemble emission.

References

(1) Schneider, C. A.; Rasband, W. S.; Eliceiri, K. W. NIH Image to ImageJ: 25 Years of Image Analysis. *Nat Methods* **2012**, *9* (7), 671–675. https://doi.org/10.1038/nmeth.2089.

(2) Fonseka, H. A.; Caroff, P.; Guo, Y.; Sanchez, A. M.; Tan, H. H.; Jagadish, C. Engineering the Side Facets of Vertical [100] Oriented InP Nanowires for Novel Radial Heterostructures. *Nanoscale Res Lett* **2019**, *14* (1), 399. https://doi.org/10.1186/s11671-019-3177-6.

(3) Mimila-Arroyo, J., Díaz-Reyes, J., and Lusson, A. Indium phosphide vapor phase epitaxy at high growth rates, growth kinetics, and characterization. *J. Appl. Phys.* **1998**, *84(3)*,1572. https://doi.org/10.1063/1.368225.
